# The Potential Role of Microsurgical Training in Robotic Surgery Education: A Prospective Simulation-Based Study

**DOI:** 10.3390/jcm15072598

**Published:** 2026-03-29

**Authors:** Alberto Bolletta, Mirco Pozzi, Davide Di Seclì, Alfredo Dente, Luigi Bonat Guarini, Stefano Bacchini, Luigi Losco, Emanuele Cigna

**Affiliations:** 1 Plastic Surgery and Microsurgery Unit, Department of Translational Research and New Technologies in Medicine and Surgery, University of Pisa, 56126 Pisa, Italy; alberto.bolletta@unipi.it (A.B.); mircovirgilio.pozzi@gmail.com (M.P.); davidedisecli@hotmail.it (D.D.S.); dente.alfredo96@gmail.com (A.D.); emanuele.cigna@unipi.it (E.C.); 2 Plastic and Reconstructive Surgery Unit, Department of Medical, Surgical and Health Sciences, University of Trieste, 34127 Trieste, Italy; luigibonat@gmail.com; 3 Plastic Surgery Unit, Department of Medicine, Surgery and Neuroscience, University of Siena, 53100 Siena, Italy; 4 Plastic Surgery Unit, Department of Medicine, Surgery and Dentistry, University of Salerno, 84084 Salerno, Italy; llosco@unisa.it

**Keywords:** microsurgery, robotic surgery, training, learning, microsurgical skills

## Abstract

**Background/Objectives**: Simulation has become an integral part of contemporary surgical training, allowing safe acquisition of technical skills with objective performance assessment. Microsurgery and robotic surgery share several technical features, including fine bimanual coordination, precise instrument control, and stereoscopic vision. This study aimed to evaluate whether a structured microsurgical course is associated with improved performance on a robotic surgical simulator and to explore its potential role within robotic training pathways. **Methods**: A prospective study was conducted between October 2022 and November 2025 at a single academic center, including 56 participants divided into three groups. Group A consisted of surgical residents attending a 3-day Basic Microsurgery Course; Group B included residents who did not undergo training during the same period; and Group C comprised experienced microsurgeons. Groups A and B performed two robotic simulation tasks at baseline (T0) and after three days (T1). Group C was assessed at T1 only as a reference benchmark. Performance was evaluated using simulator-derived metrics. Statistical analysis was performed using paired and unpaired t-tests. **Results**: Group A showed significant improvement across several performance parameters following training, whereas no comparable changes were observed in Group B. At T1, Group A demonstrated better performance than Group B in multiple metrics. Group C achieved the highest scores and was considered a reference group. **Conclusions**: Structured microsurgical training was associated with improved performance in a robotic simulation setting. These findings suggest that microsurgical skills may be transferable to robotic tasks and may contribute to the early phases of robotic skill acquisition. Further studies are required to assess their impact in clinical practice.

## 1. Introduction

Surgical training has always been a great challenge in surgery schools. For a long time, training in surgery has based its principles on practical experience with real-life models as experience itself helps with learning. This is the well-known “see one, do one, teach one” model, which has been the basis for surgical training for years; however, over time, this model proven its weakness, as it depends on direct training on real patients [1,2,3,4]. Surgery residents have stated that the are not completely confident in performing surgical procedures safely on their own when basing their experience on this kind of training.

Nowadays, a surgeon’s level of expertise has to be objectively demonstrated, and this requires multiple types of training and simulation [4,5,6,7,8,9]. Several studies have demonstrated the importance of simulation in learning surgical procedures, which enables residents to practice independently without the risk of causing real harm; indeed, observing a procedure is fundamentally different from performing it firsthand, and we retain only 20% of what we see but 70% of what we do. This is why simulations have become increasingly popular in surgical training, with microsurgery and robotic surgery being two of the most prominent examples [7,8,9,10].

Microsurgery and robotic surgery are based on preclinical practice: numerous courses exist, based on different levels and using different models, and taking part in these training courses allows surgeons to learn new skills and improve their abilities. First surgical procedures should be done not on real patients but on alternative models [10,11,12,13,14,15]. For these reasons, training centers have standardized their training courses to allow the acquisition of skills with graduality, starting with artificial models (gauze, latex tube), continuing with ex vivo models and concluding with animal models (rats).

These courses enable surgeons to develop and refine essential microsurgical skills, including visuo-spatial abilities, precision in movement, dexterity, and the ability to work with binocular vision. These competencies are particularly beneficial for young microsurgeons in tasks such as vessel dissection, precise suture placement during anastomosis, and the delicate manipulation of tissues and instruments under a microscope. Microsurgery has its own learning curve, and simulations can be used by microsurgeons to objectively evaluate their training before clinical practice; these skills are often significantly improved even after a single-day training course in microsurgery [16,17,18,19].

As the same abilities are required in robotic surgery, some authors started to notice these similarities and conducted a prospective study comparing microsurgeons’ and general surgeons’ ability to use a robotic surgery simulator, and demonstrated better results for microsurgeons than general surgeons. Therefore, a microsurgery training course could be an effective initial step in approaching robotic surgery. To date, only one original article has demonstrated a positive correlation between these two techniques; in that study a group of surgeons inexperienced in microsurgery was compared with a control group of experienced microsurgeons; however, the potential correlation between improvement in performance in conventional robotic surgery and microsurgical training has not yet been investigated [20].

Given the limited literature and data in this field, and recognizing the growing importance of simulation in surgical training, it is generally accepted that increased surgical exposure improves technical performance; however, it remains unclear whether specific training modalities confer a distinct advantage in robotic skill acquisition. Microsurgical training involves fine bimanual coordination, tremor control, and the ability to operate under magnification, all of which are also required in robotic surgery. For this reason, microsurgery may represent a form of targeted psychomotor and visuospatial conditioning rather than simply an additional training experience. However, the extent to which these skills translate into measurable improvements in robotic simulation has not been clearly established.

The aim of this study was therefore to evaluate whether a structured basic microsurgical course is associated with measurable improvement in robotic surgical simulator performance. This question is clinically relevant, as the potential integration of microsurgical training into robotic surgery curricula may contribute to improving the learning curve and accelerating skill acquisition in trainees learning to use robotic platforms.

## 2. Materials and Methods

A prospective study was conducted between October 2022 and November 2025 at the Department of Plastic Surgery and Microsurgery of the University Hospital, Pisa, Italy, involving 56 participants divided into three groups.

Group A consisted of surgery residents with no prior experience in microsurgery, robotic surgery or laparoscopic surgery, enrolled in a Basic Microsurgery Course. Group B, the control group, included surgery residents with no prior experience in microsurgery, robotic surgery or laparoscopic surgery, not enrolled in the Basic Microsurgery Course. Group C comprised experienced microsurgeons.

Participants were allocated to Group A or Group B based on enrollment in the Basic Microsurgery Course. All residents who had registered for the course were included in Group A, as they were scheduled to attend the training independently of the study. No randomization was performed.

Both Groups A and B consisted of residents without prior experience in microsurgery, laparoscopy, or robotic surgery. These two groups were not matched beyond baseline performance on the robotic simulator, which was comparable at T0. No additional comparisons between groups were performed outside the simulator-based assessment.

**Figure 1:**
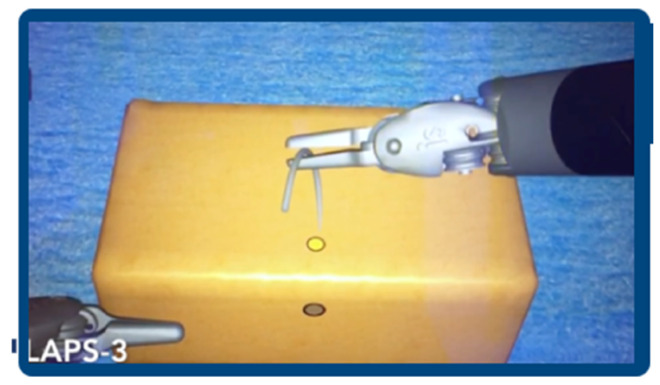
Robotic Simulator Exercise, Suture Sponge 1.

**Figure 2:**
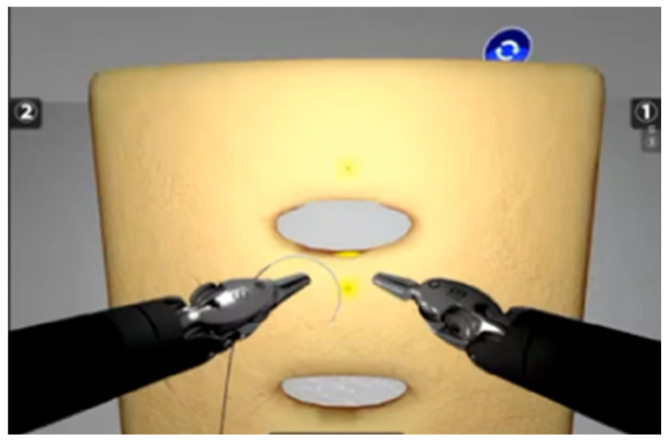
Robotic Simulator Exercise, Horizontal Defect Suturing 1 (HDS1).

All participants were informed about the exercises and the parameters that would be analyzed. Groups A and B underwent an initial test at Time 0 (T0) using the Da Vinci Skills Simulator (dVSS; Intuitive Surgical, Inc., Sunnyvale, CA, USA)performing two exercises:

- Suture Sponge 1 (SS1), designed to improve precision and needle handling in a deformable object (Figure 1).
- Horizontal Defect Suturing 1 (HDS1), which focused on tying a simple surgical knot in a horizontal wound, simulating robotic suturing techniques (Figure 2).

After the T0 test, Group A attended a three-day Basic Microsurgery Course where they took part in theoretical lessons and practical sessions; Group B underwent a 72 h rest period without training. After this interval, both groups underwent a final test at Time 1 (T1), repeating the same SS1 and HDS1 exercises on the DaVinci Skills Simulator. Additionally, Group A completed a questionnaire, consisting of 9 items, assessing perceived similarities and differences between microsurgery and robotic surgery; the questionnaire was specifically designed for this study and was not subjected to either validation or pilot testing. Only participants in Group A completed the questionnaire, as they were the only ones who attended the course, and the questionnaire was based on subjective differences between microsurgical activity and robotic simulator activity.

Group C, composed of experienced microsurgeons, performed the same exercises at T1.

The performance parameters for Groups A and B were recorded by the simulator and compared between T0 and T1 (Table 1).

**Figure 3:**
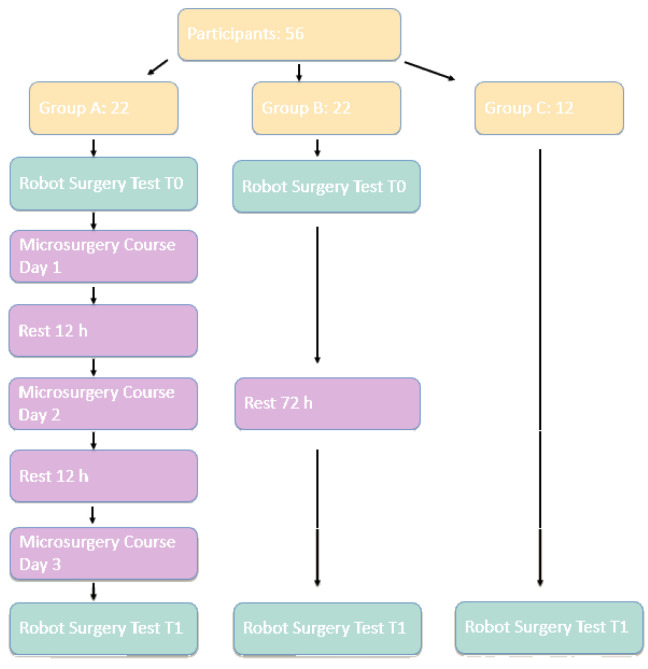
Flow diagram illustrating the study design.

For Group C, the same parameters were analyzed at T1. Figure 3 shows a flow diagram of the study.

Statistical analysis was performed using paired and unpaired t-tests, as appropriate. All values were reported as the mean ± SD, and differences between groups were analyzed using a t-test, with statistical significance set at *p* < 0.05.

Ethical review and approval were not required for this study, as this was a simulation-based educational study involving adult healthcare professionals, with no patients enrolled, no clinical interventions, and no collection of identifiable patient data.

All participants were informed about the study and provided voluntary consent to participate.

## 3. Results

The results of this study were collected between October 2022 and November 2025, with a total of 56 participants enrolled. Group A consisted of 22 plastic surgery residents. Group B included 22 plastic surgery residents. Participants in both groups were between 25 and 35 years old. Group C comprised 12 experienced microsurgeons, ranging in age from 40 to 55 years.

The results are presented in Table 2 and Table 3, expressed as the mean ± SD, and data analysis revealed significant differences between the groups.

In order to evaluate the effect of microsurgical training on the performance of the three groups on the robotic surgery simulator, firstly, parameters of both exercises performed by Group A were analyzed, comparing the results before and after the 3-day course. A significant improvement was shown in overall score (27.16 ± 25.40 vs. 58.94 ± 17.60, *p*: 0.020), time to complete exercise (695.74 ± 274.61 vs. 339.87 ± 80.61, *p*: 0.0139), economy of motion (550.00 ± 162.33 vs. 342.24 ± 93.20, *p*: 0.014), instrument out of view (0.92 ± 1.27 vs. 0.37 ± 0.65 *p*: 0.046) and management of work space (5.92 ± 1.43 vs. 4.46 ± 1.02, *p*: 0.0499) for the SS1 exercise. A significant improvement was also shown in overall score (10.90 ± 10.12 vs. 31.71 ± 24.07, *p*: 0.048) and time to complete exercise (505.71 ± 264.83 vs. 265.57 ± 76.52, *p*: 0.049) for the HDS1 exercise.

The same data were collected for Group B participants, showing no significant improvement after 72 h of resting.

Moreover, the analysis compared the data between Groups A and B: the results showed no statistically significant differences at T0, but showed significant differences at T1 in overall score (58.94 ± 17.60, *p*: 0.013), time to complete exercise (339.87 ± 80.61 vs. 549.52 ± 238.90, *p*: 0.019), instrument out of view (0.37 ± 0.65 vs. 3.30 ± 5.54, *p*: 0.046) and management of workspace (4.46 ± 1.02 vs. 6.41 ± 1.35, *p*: 0.003) for the SS1 exercise. Significant differences were also obtained in time to complete exercise (265.57 ± 76.52 vs. 518.18 ± 232.20, *p*: 0.005) for the HSD1 exercise.

Finally, comparing the data between groups, A, B and C, a significant difference between the results was shown for almost all the parameters analyzed (Table 4).

Only participants in Group A, who had attended the Basic Microsurgery Course, completed the questionnaires; specifically, 21 out of 22 participants responded. The results, expressed as absolute numbers and percentages, are reported in detail in Table 5.

## 4. Discussion

Given the limited literature and data in this field, and recognizing the growing importance of simulation in surgical training, it is generally accepted that increased surgical exposure improves technical performance; however, it remains unclear whether specific training modalities confer a distinct advantage in robotic skill acquisition. Microsurgical training involves fine bimanual coordination, tremor control, and the ability to operate under magnification, all of which are also required in robotic surgery. For this reason, microsurgery may represent a form of targeted psychomotor and visuospatial conditioning rather than simply an additional non-specific training experience.

The three-day Basic Microsurgery Course is built around four core exercises: suturing on gauze and latex, anastomosis on silicone tubes, and anastomosis on ex vivo animal vessels. The course combines short theoretical sessions with hands-on training and is designed to improve visuospatial skills, hand–eye coordination, and economy of movement under magnification. It also introduces practical principles that trainees can immediately transfer to simulation-based robotic tasks. Both microsurgery and robotic surgery require structured training before clinical application, and standardized simulation programs remain essential to develop safe and reproducible fundamentals. Given the known overlap between microsurgery and robotic surgery [7], and the need to optimize simulation pathways for trainees, we aimed to evaluate how a microsurgery course influences robotic simulator performance and how participants perceive similarities between the two techniques.

In our study, Groups A and B were compared with regard to the role of microsurgery training in influencing robotic skills. Participants of both groups underwent an initial and a final test on a robotic surgery simulator, completing two exercises, SS1 and HDS1. Both exercises were similar to exercises performed during the Basic Microsurgery Course (suture on gauze and a latex tube and then on ex vivo models could reproduce the movements and skills required by the two exercises on the robotic surgery simulator). Comparing the performance of the two groups before and after the Basic Microsurgery Course, Group A showed an improvement in total score, time to complete exercise and management of workspace in the traditional robotic surgery simulation; in practical terms, after the Basic Microsurgery Course, participants were faster and handled the operative workspace more effectively during robotic simulation. Because microsurgery and robotic surgery share key technical elements, such as precise bimanual movements, fine instrument control, and stereoscopic depth perception, and attendees also demonstrated an improvement in the economy of movement and in instrument handling when working outside the direct field view. These improvements were not significant in Group B, which, after the first simulation session, underwent a resting period before repeating the traditional robotic surgery simulation.

In our study we also evaluated if long-term microsurgery experience could influence robotic surgery simulation performance. To do so, a group of experienced microsurgeons underwent the same robotic surgery simulation tests at T1, and we compared the results of Group C with those of Groups A and B. Expert microsurgeons showed better results for total score, time to complete exercise, management of workspace and economy of movements. They also performed better in error-related metrics, including drops and missed targets, which did not improve meaningfully after the basic course in Group A. This finding is consistent with what we see in practice: a basic course improves core mechanics and efficiency, while advanced training and operating room experience refine precision, anticipation, and error avoidance; however, Group C was included as a reference group to provide a performance benchmark and was not intended for direct comparison with Groups A and B, given the differences in age and general surgical experience. In addition to microsurgical expertise, overall surgical experience itself may represent a potential source of bias influencing performance outcomes.

The questionnaire supported the technical overlap between microsurgery and robotic surgery. Most participants found similarities in the sitting position, ergonomics, position of forearms, workspace, tissue manipulation, wrist movements, and binocular vision. However, in robotic surgery, the binocular visualization, similar to that provided by the microsurgical exoscope, likely makes visual accommodation less demanding compared with a conventional operating microscope [21]. They also perceived similarities between robotic joysticks and microsurgical instruments, particularly needle holders and forceps. Ergonomics remains central in both settings, from stable upper-limb support to consistent head and visual alignment for stereoscopic work.

Among the questionnaire results, responses to question no. 6 revealed a significant difference in the perceived difficulty of the task, attributed to the absence of haptic feedback in traditional robotic systems. The Da Vinci Skills Simulator used in our study does not provide such feedback [22,23].

Statistical analysis was performed using paired and unpaired t-tests, as appropriate, to evaluate within-group changes over time and between-group differences at each time point. Given the exploratory design of the study and the relatively limited sample size, this approach was considered appropriate to provide a clear and direct interpretation of the main outcomes across the selected simulator parameters. The analysis was therefore focused on predefined comparisons of clinical and educational relevance rather than on a global modeling approach.

We acknowledge that this approach may increase the risk of a type I error when multiple endpoints are analyzed. However, the consistency of the observed improvements across several parameters in the training group, together with the absence of a similar pattern in the control group, supports the overall robustness of the findings.

Future studies with larger cohorts may benefit from the use of more comprehensive statistical models, such as repeated-measures or mixed-effects analyses, to further refine the evaluation of changes over time and interactions between groups.

Robotic assistance in microsurgery has evolved from experimental studies to clinical implementation across a spectrum of reconstructive indications, including lymphatic surgery, free tissue transfer, and nerve coaptation. It has been demonstrated that microsurgical robotic systems can support microvascular anastomosis while enabling objective performance assessment through structured scoring tools, highlighting measurable improvements in technical execution and operative efficiency with repeated exposure [24]. The emergence of robotic platforms has introduced a paradigm shift, offering motion scaling, tremor filtration, enhanced visualization, and ergonomic advantages that may mitigate some of the inherent limitations of human performance during delicate microvascular and neural procedures. Preclinical investigations involving heterogeneous cohorts of surgeons with varying experience levels have shown that robotic platforms facilitate rapid improvement in performance metrics, including operative time and structured skill assessment scores, suggesting that robotic proficiency may be attainable across different training backgrounds. An additional noteworthy observation relates to the influence of prior surgical experience on robotic microsurgery skill acquisition. Although baseline experience correlates with early performance metrics, reflecting transferable fundamental surgical abilities, this effect diminishes with successive training sessions, suggesting that robotic proficiency may ultimately become independent of traditional microsurgical expertise [25]. Subsequent clinical experience with dedicated microsurgical robots, particularly the Symani Surgical System, has confirmed feasibility and safety in human procedures while revealing a characteristic learning curve associated with integration into routine surgical workflow. In early case series utilizing the Symani Surgical System^®^, robotic anastomotic times were initially longer than hand-only techniques but declined consistently with experience, ultimately approaching equivalence in selected procedures such as lymphovenous anastomosis. This temporal convergence underscores a familiar phenomenon observed during the introduction of novel surgical technologies, where early inefficiency reflects system familiarization rather than intrinsic limitations of the platform. Moreover, high anastomotic patency rates and the successful completion of many reconstructive procedures demonstrate that robotic assistance can be safely integrated into clinical practice. Similarly to traditional robotic surgery, in robotic microsurgery, the absence of haptic feedback, highlighted in early clinical experience, introduces potential risks related to excessive force application and underscores the need for adapted handling strategies during robotic manipulation [26]. While this study focuses on conventional robotic simulation, it is plausible that the overlap between microsurgical skills and robotic platforms may be even more pronounced in the setting of robotic microsurgery, where technical demands more closely resemble those of traditional microsurgery. From this perspective, the integration of standardized microsurgical training within a robotic microsurgery training pathway may result in further improvements in performance metrics over time. This hypothesis, however, remains to be formally tested. 

At the same time, it should be noted that surgeons performing robotic microsurgery are often already experienced microsurgeons, which may limit the possibility of studying truly naïve populations and introduce potential selection bias.

It would be of interest for future studies to include robotic systems equipped with haptic feedback to assess its potential impact. Additionally, it would be valuable to conduct a reverse study, namely, to evaluate whether a standardized basic training course in robotic surgery could improve microsurgical performance outcomes.

Further studies with more participants and with an analysis of more parameters could lead to a more accurate evaluation of how microsurgical training can help in robotic surgery.

## 5. Conclusions

Surgical training remains a demanding process, and simulation has become an integral component of contemporary training pathways. It provides a controlled environment in which residents can develop fundamental technical skills and monitor their progression through objective parameters. Microsurgery and robotic surgery share several core technical features, including fine bimanual coordination, precise instrument control, and stereoscopic vision.

In this study, participation in a structured microsurgical course was associated with improved performance on a robotic surgical simulator. These findings suggest that microsurgical training may provide a form of targeted psychomotor conditioning, rather than simply increasing overall surgical exposure, and may facilitate the early phases of robotic skill acquisition.

From a training perspective, this may have practical implications in structuring educational pathways, particularly in the initial phase of robotic training, where acquisition of basic technical skills remains a critical step.

These results should, however, be interpreted with caution. The study design does not allow conclusions on causality, and the findings are limited to a simulation environment. Further studies are needed to assess whether this effect has a measurable impact in clinical practice and to better define its role within surgical training pathways.

## Figures and Tables

**Table 1:** Parameters analyzed by DaVinci Skills Simulator.

SS1	HDS 1
Overall score %	Total score
Time to complete exercise (s)	Time to complete
Economy of motion	
Instrument collision	
Excessive instrument force	
Instrument out of view	
Management of workspace	
Drops	
Missed target	

SS1: Suture Sponge 1; HDS: Horizontal Defect Suturing 1.

**Table 2:** Suture Sponge 1 exercise results.

	Group A		Group B		Group C
	T0	T1	T0	T1	T1
**Overall score %**	27.16 ± 25.40	58.94 ± 17.60	23.18 ± 20.57	33.08 ± 20.56	72.57 ± 15.37
**Time to complete exercise (s)**	695.74 ± 274.61	339.87 ± 80.61	609.18 ± 249.24	549.52 ± 238.90	265.48 ± 58.84
**Economy of motion**	550.00 ± 162.33	342.24 ± 93.20	597.34 ± 248.76	498.42 ± 222.36	230.18 ± 156.96
**Instrument collision**	17.14 ± 16.30	4.57 ± 4.43	14.73 ± 10.46	11.91 ± 10.89	4.30 ± 5.02
**Excessive instrument force**	4.44 ± 6.83	2.22 ± 2.86	7.13 ± 6.63	4.36 ± 4.68	1.15 ± 1.61
**Instrument out of view**	0.92 ± 1.27	0.37 ± 0.65	3.05 ± 4.42	3.30 ± 5.54	1.14 ± 1.99
**Management of workspace**	5.92 ± 1.43	4.46 ± 1.02	5.78 ± 1.48	6.41 ± 1.35	4.56 ± 1.50
**Drops**	2.43 ± 2.64	0.43 ± 0.53	1.82 ± 2.23	1.18 ± 1.54	0.33 ± 0.52
**Missed target**	29.29 ± 33.39	16.57 ± 14.59	25.55 ± 12.79	27.82 ± 18.30	13.17 ± 7.70

T0: Time 0; T1: Time 1.

**Table 3:** Horizontal Defect Suturing 1 exercise results.

	Group A		Group B		Group C
	T0	T1	T0	T1	T1
**Total score**	10.90 ± 10.12	31.71 ± 24.07	6.09 ± 10.52	15.66 ± 13.74	49.10 ± 11.04
**Time to complete**	505.71 ± 264.83	265.57 ± 76.52	691.73 ± 287.29	518.18 ± 232.20	222.50 ± 65.35

T0: Time 0; T1: Time 1.

**Table 4:** *p* values of differences in results. *P* values marked with (*) are <0.05.

Group A		*p* Value, Group A at T0 and T1	*p* Value, Group B at T0 and T1	*p* Value, Groups A and B at T0	*p* Value, Groups A and B at T1	*p* Value, Groups A, B, and C at T1
**SS1**	**Overall Score**	0.020 *	0.272	0.730	0.013 *	0.008 *
	**Time**	0.013 *	0.573	0.510	0.019 *	0.005 *
	**Economy of motion**	0.014 *	0.337	0.630	0.056	<0.001 *
	**Instrument collision**	0.089	0.543	0.730	0.066	0.007 *
	**Excessive instrument force**	0.448	0.273	0.420	0.244	0.022 *
	**Instrument out of view**	0.046 *	0.018 *	0.046 *	0.046 *	0.078
	**Management of workspace**	0.0499 *	0.307	0.840	0.003 *	<0.001 *
	**Drops**	0.0968	0.446	0.620	0.160	0.037 *
	**Missed Targets**	0.383	0.739	0.780	0.170	0.019 *
**HDS1**	**Overall Score**	0.048 *	0.053	0.350	0.143	<0.01 *
	**Time**	0.0496 *	0.135	0.180	0.005 *	<0.01 *

SS1: Suture Sponge 1; HDS: Horizontal Defect Suturing 1.

**Table 5:** Questionnaire (9 questions) on perceived similarities and differences between microsurgery and robotic surgery. The questionnaires were completed by participants in Group A at time T1, and the results are expressed as percentages of the total Group A cohort.

QUESTIONS		
	YES	NO
1. Between microsurgery and robotic surgery do you notice a similarity in the ergonomics of posture?	18 (85.7%)	3 (14.3%)
2. Between microsurgery and robotic surgery do you notice a similarity in sitting posture?	21 (100%)	0 (0%)
3. Between microsurgery and robotic surgery do you notice a similarity in forearm position?	18 (85.7%)	3 (14.3%)
4. Between microsurgery and robotic surgery do you notice a similarity in working space?	12 (57.1%)	9 (42.9%)
5. Do you notice a similarity between joysticks and microsurgical instruments?	18 (85.7%)	3 (14.3%)
6. Is there the same difficulty between microsurgery and robotic surgery due to the lack of force feedback?	7 (33.3%)	14 (66.7%)
7. Between microsurgery and robotic surgery do you notice a similarity in tissue manipulation with joysticks and microsurgical instruments?	15 (71.4%)	6 (28.6%)
8. Between microsurgery and robotic surgery do you notice a similarity in wrist movement?	18 (85.7%)	3 (14.3%)
9. Between microsurgery and robotic surgery do you notice a similarity in the binocular vision system?	18 (85.7%)	3 (14.3%)

## Data Availability

The original contributions presented in this study are included in the article. Further inquiries can be directed to the corresponding author.
